# Large measles epidemic in the Netherlands, May 2013 to March 2014: changing epidemiology

**DOI:** 10.2807/1560-7917.ES.2017.22.3.30443

**Published:** 2017-01-19

**Authors:** Tom Woudenberg, Rob S. van Binnendijk, Elisabeth A. M. Sanders, Jacco Wallinga, Hester E. de Melker, Wilhelmina L. M. Ruijs, Susan J. M. Hahné

**Affiliations:** 1 National Institute for Public Health and the Environment (RIVM), Bilthoven, the Netherlands; 2 University Medical Center Utrecht, Utrecht, the Netherlands; 3 Leiden University Medical Center, Leiden, the Netherlands

**Keywords:** measles, outbreaks, epidemiology, emerging or re-emerging diseases, surveillance, vaccine-preventable diseases

## Abstract

Since the early 1990s, the Netherlands has experienced several large measles epidemics, in 1992–94, 1999–2000 and in 2013–14. These outbreaks mainly affected orthodox Protestants, a geographically clustered population with overall lower measles-mumps-rubella first dose (MMR-1) vaccination coverage (60%) than the rest of the country (> 95%). In the 2013–14 epidemic described here, which occurred between 27 May 2013 and 12 March 2014, 2,700 cases were reported. Several control measures were implemented including MMR vaccination for 6–14-month-olds and recommendations to reduce the risk in healthcare workers. The vast majority of reported cases were unvaccinated (94%, n = 2,539), mostly for religious reasons (84%, n = 2,135). The median age in the epidemic was 10 years, 4 years older than in the previous epidemic in 1999–2000. A likely explanation is that the inter-epidemic interval before the 2013–2014 epidemic was longer than the interval before the 1999–2000 epidemic. The size of the unvaccinated orthodox Protestant community is insufficient to allow endemic transmission of measles in the Netherlands. However, large epidemics are expected in the future, which is likely to interfere with measles elimination in the Netherlands and elsewhere.

## Introduction

Measles is a highly contagious infectious disease caused by the measles virus. It can lead to serious illness, life-long complications and death [1]. Measles vaccination programmes have contributed to a steep decline in the number of infections and deaths, but in 2014 measles still caused an estimated 114,900 deaths worldwide, mostly in low-income countries [2]. Case fatality is reported to be up to 6% in developing countries and is especially high in infants and young children [3].

In the Netherlands, a single-dose measles vaccination programme was introduced within the national immunisation programme (NIP) in 1976 for all infants at 14 months of age. Since 1987, a two-dose programme using measles-mumps-rubella (MMR) vaccine has been offered at 14 months and 9 years of age. Vaccine coverage of the first dose of MMR vaccination has been above 95% for 20 years [4]. Coverage for two doses at the age of 10 years has been around 93% for 10 years. Introduction of measles vaccination in the Dutch NIP resulted in a large decrease in the number of reported cases [5]. However, epidemics still occur due to sociogeographically clustered individuals who refrain from vaccination. A large measles epidemic occurred in 1999–2000 with 3,292 reported cases, most of whom were unvaccinated (94%) and belonged to the orthodox Protestant community (83%) [6]. Between 2001 and 2012 the incidence of measles was lower than the five cases per million set as a target by the World Health Organisation (WHO) in 2010 [7], except for 2008 when the incidence was seven per million, due to an outbreak in individuals with anthroposophic beliefs [8].

The orthodox Protestant population comprises around 1% of the total population in the Netherlands [9]. Vaccine coverage in these communities is around 60% on average, but varies widely between churches, with coverage ranging from less than 30% among members of the most orthodox churches to vaccination rates comparable to the rest of the Netherlands in the least traditional churches [10]. In general, orthodox Protestants form close-knit communities. The majority of them, ca 75%, live geographically clustered in the region known as the Bible belt. In this region, stretching from the south-west to the north-east of the country, 29 municipalities have MMR vaccination coverage of less than 90% [11]. Children in these communities often attend orthodox Protestant primary and secondary schools. Some of these schools are known to have an MMR-1 and diphtheria-tetanus-pertussis vaccination coverage below 15% [12]. A serological survey carried out in 2006–2007 confirmed a high risk of a large measles epidemic in these communities [13]. The seroprevalence was especially low in children 1–4 years of age (36%) and 5–9 years of age (63%).

The most recent epidemic started in May 2013 when two unvaccinated children attending an orthodox Protestant school were reported to have measles [14]. In response to the subsequent outbreak, on 17 June 2013 a national outbreak management team (OMT) advised early MMR vaccination for infants aged 6–14 months living in municipalities with MMR-1 vaccination coverage < 90% [15]. Infants of this age are too young to have been vaccinated in the regular schedule, but have lost their maternal antibodies against measles [16] and are at the highest risk of complications [17]. Parents of eligible infants were contacted directly and invited to this additional MMR vaccination (MMR-0 for 6–11 month-olds) or early (MMR-1 for 12–14 month-olds). This intervention was implemented between July 2013 and February 2014. In total, 5,800 infants out of 10,097 (57%) received an early MMR vaccination before 14 months of age.

Furthermore, the OMT advised communication via the media that children and teenagers up to 19 years of age were entitled to receive a free catch-up MMR vaccination. This was also communicated through a newspaper and family magazines widely read by orthodox Protestants, even though previous research showed low acceptance of catch-up vaccination among this group [18].

The OMT also advised assessment of the immune status of healthcare workers (HCW) and provision of additional MMR vaccination when required [15]. HCW who were born before 1965 or had been vaccinated twice were considered to be protected, and all other HCW were advised to complete their MMR vaccination schedule. Letters were posted to all academic and community hospitals explicitly requesting them to bring this advice to the attention of the infection control committee.

Here we describe the epidemiology of the 2013–2014 measles epidemic in the Netherlands and compare it with the previous epidemic in 1999–2000.

## Methods

### Notification of measles

Measles is a mandatory notifiable disease in the Netherlands. Physicians and laboratories are required to report cases to Municipal Health Services (MHS). Directors of schools and day care centres are required to report rash clusters in their institutions to MHS. For every reported case, a MHS physician or nurse must complete a standardised questionnaire. The questionnaire covers, among others things, demographic characteristics, disease onset dates, hospitalisation, possible source, presence of complications, probable place of infection, vaccination status and reasons for non-vaccination. A possible source of infection is defined as contact with another reported case 7 to 21 days before the onset of the rash. Reasons for non-vaccination are pre-specified in the questionnaire and cases can be categorised into one of the following risk groups: orthodox Protestant, individual with anthroposophic beliefs, individual with a critical attitude towards vaccination, unknown or none of the pre-specified risk groups. The National Institute of Public Health and the Environment (RIVM) maintains an electronic web-based register for notifications by the MHS.

### Case definition

Clinical measles is defined as fever and a maculopapular rash accompanied by at least one of the following three symptoms: cough, coryza or conjunctivitis. Cases of measles are defined as clinical measles in a person with laboratory-confirmed measles virus infection and/or an epidemiological link to a laboratory-confirmed case. A case is epidemiologically linked if the individual had contact with a laboratory-confirmed case in the 3 weeks before onset of disease. Laboratory confirmation is based on positive measles-specific IgM serology and/or detection of measles virus RNA by PCR in a throat swab, oral fluid or urine specimen [19]. Physicians were advised to rapidly diagnose individuals presenting with severe illness, which was mostly done by testing for measles-specific IgM. In other cases, the use of less invasive sampling of oral fluid was recommended, which comprised 60% of the specimens forwarded to the national laboratory for PCR testing; the remainder were throat swabs or urine specimens. The majority of PCR-positive specimens were selected for genotyping using primers amplifying the N-terminal 450-nt fragment of the measles nucleocapsid gene, according to WHO-approved sequencing methods for genotyping as previously described [20]. In case of successful and complete sequencing results, genotypes were generated and representative sequences were reported to the WHO Measles Nucleotide Surveillance (MeaNS) database.

From mid-July 2013 onwards, MHS located in the Bible belt were advised by the RIVM to limit the use of laboratory diagnostics of measles to cases with complications, vaccinated cases, cases in newly affected schools, villages or risk groups and cases that were reported by general practitioners without an epidemiological link.

Our analyses included all cases reported between 27 May 2013 and 12 March 2014, respectively the first and last date with a case laboratory-confirmed with the predominant outbreak strain. Imported cases and cases with a genotype or strain other than the outbreak strain were excluded. Cases that were epidemiologically linked to excluded cases were also excluded.

Population data was retrieved from Statistics Netherlands. Vaccine coverage by municipality and postal code area was available from the national vaccination register. Proportions were compared using the chi-squared test or Fisher’s exact test. The age distributions of both epidemics were compared using the Kolmogorov-Smirnov test. To test differences in medians we used Mood’s median test. All analyses were performed using R software, version 3.1.0. Maps were created with ArcGIS version 10.2.2.

## Results

### Outbreak description

Overall, 2,766 measles cases were reported between 27 May 2013 and 12 March 2014. Molecular typing of the outbreak strain showed a genotype D8 measles virus (strain MVs/Alblasserdam.NLD/22.13, WHO/MeaNS Id 50730, GenBank Id KM066606), with a sequence indistinguishable from the strain that was first identified in the United Kingdom (UK) in 2012 (MVs/Taunton.GBR/27.12, WHO/MEANS Id 23447, GenBank Id JX984461). Two per cent (n = 66) of the cases were excluded because they had a different genotype (n = 11) or were imported (n = 25). Epidemiologically linked to these different genotypes and importations were 20 and 10 cases, respectively. Of the 11 different genotypes found, 10 were genotype B3 and one genotype H1. We included the remaining 2,700 cases in our analyses.

**Figure 1:**
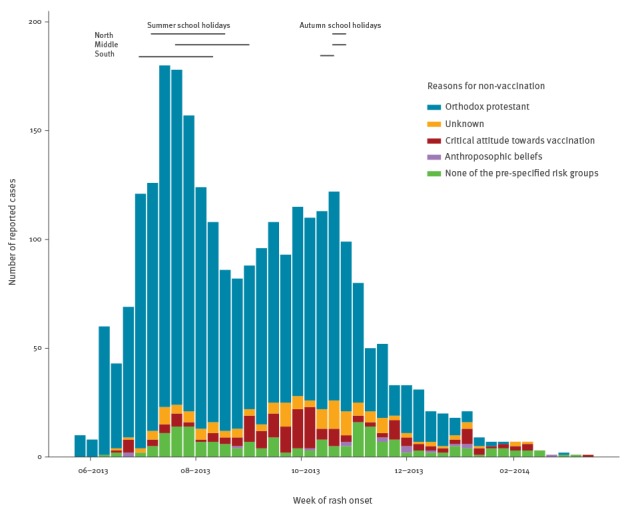
Reported measles cases by risk group and week of rash onset, the Netherlands, reported between 27 May 2013 and 12 March 2014 (n = 2,700)

The first two cases were reported on 27 May 2013 in two unvaccinated children attending the same orthodox Protestant primary school. These children had not travelled abroad and the source of their measles infection was unknown. The epidemic peaked in the second week of July 2013 with 180 reported cases, with a subsequent rapid decline during school holidays in July and August 2013 (Figure 1). Coinciding with the new school year, from September 2013 onwards, reported cases increased until another peak of 122 cases occurred in the third week of October. Subsequently, the number of cases per week declined. The last case was reported on 12 March 2014.

If the first day of rash onset was not available, the date of the onset of the prodromal phase + 3 days was used [17]. Horizontal lines indicate the timing of school holidays, which differ by region.

The vast majority of reported cases were unvaccinated (94%, n = 2,539) (Table), mostly based on religious grounds (84% of unvaccinated cases, n = 2,135). Others who refrained from vaccination were people who had anthroposophic beliefs (1%, n = 16), had a critical attitude towards vaccination (7%, n = 172) or had other reasons to refrain from vaccination (4%, n = 108). Of vaccinated cases (n = 141), 89% (n = 125) had been vaccinated once, 11% had been vaccinated twice (n = 15), and one individual had been vaccinated three times (0.1%) (Table). Sixty-eight per cent (n = 85) of the 125 once-vaccinated cases were between 14 months and 8 years of age, and of those, 49% (n = 61) were between 4 and 8 years of age. The majority of the 16 twice-vaccinated cases were older than 18 years of age (n = 13).

**Table:** Reported measles cases by vaccination status (n = 2,700), hospitalisation (n = 2,677) and complications (n = 2,581) during a measles epidemic, the Netherlands, May 2013 – March 2014

No. (%)^a^ reported cases by age group								
	**0–13** ** months **	**14–48** ** months **	**4–8** ** years **	**9–17** ** years **	**18–40** ** years **	**> 40** ** years **	**Total **	**p value^b^**
**Vaccination status**	**n = 78**	**n = 260**	**n = 824**	**n = 1,268**	**n = 226**	**n = 44**	**n = 2,700**	
Unvaccinated	75 (96)	236 (91)	760 (93)	1246 (99)	183 (81)	39 (89)	2,539 (94)	NA
Once	3 (4)	24 (9)	61 (7)	16 (1)	20 (9)	1 (2)	125 (5)	NA
Twice or more	0 (0)	0 (0)	0 (0)	3 (0)	13 (6)	0 (0)	16 (1)	NA
Unknown	0 (0)	0 (0)	3 (0)	3 (0)	10 (4)	4 (9)	20 (1)	NA
**Complication ^c^**	**(n = 75)**	**(n = 247)**	**(n = 787)**	**(n = 1,208)**	**(n = 221)**	**(n = 43)**	**(n = 2,581)**	
All complications	12 (16)	41 (17)	108 (14)	111 (9)	17 (8)	7 (16)	296 (11)	< 0.01
Pneumonia^d^	8 (11)	23 (9)	54 (7)	61 (5)	12 (5)	3 (7)	161 (6)	0.07
Otitis media^d^	4 (5)	16 (6)	48 (6)	41 (3)	4 (2)	0 (0)	113 (4)	< 0.01
Encephalitis^d^	0 (0)	0 (0)	1 (0)	1 (0)	0 (0)	0 (0)	2 (0)	NS
Dehydration/diarrhoea^d^	0 (0)	4 (2)	8 (1)	12 (1)	3 (1)	3 (7)	30 (1)	0.07
Other^de^	0 (0)	0 (0)	3 (0)	2 (0)	1 (0)	1 (2)	7 (0)	NS
**Hospitalisation status^f^**	**(n = 77)**	**(n = 257)**	**(n = 819)**	**(n = 1,254)**	**(n = 226)**	**(n = 44)**	**(n = 2,677)**	
Hospitalised	8 (10)	24 (9)	51 (6)	55 (4)	32 (14)	11 (25)	181 (7)	< 0.01

NA: not applicable; NS: not significant.

^a^ Proportion of the number of reported cases by age group.

^b^ Fisher’s exact test or chi-squared test.

^c^ Information on complications was unknown for 119 cases.

^d^ Individual cases could have multiple complications. For example, five cases had both otitis media and pneumonia

^e^ Other complications comprised bilateral striatal necrosis (n = 1), hepatitis (n = 1), keratitis (n = 1), stomatitis (n = 1), tonsillitis (n = 2), and transverse myelitis (n = 1). Respiratory infections other than pneumonia were not included under ‘other complications’.

^f^ Information on hospitalisation was unknown for 23 cases.

**Figure 2:**
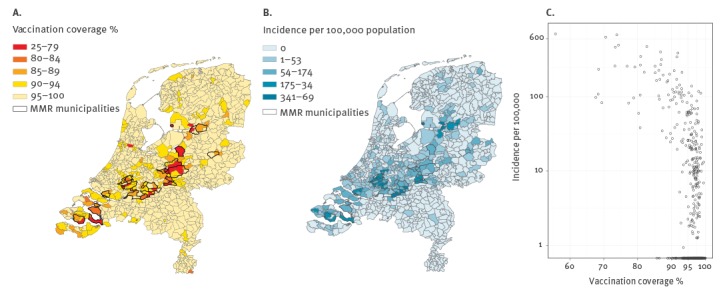
A) MMR-1 vaccination coverage combined for birth cohorts 2011/2010/2009 at the age of 2 years, by three-digit postal code. B) Measles incidence from reported cases from May 2013 until March 2014 (n = 2,689), by three-digit postal code in the Netherlands. C) Scatterplot (log-scale) of three-digit postal code areas vaccination coverage and reported measles incidence.

The epidemic mainly affected low-vaccination-coverage areas. Nearly half of reported cases (49%) occurred in the 29 municipalities with vaccination coverage below 90% (range 60– 90%). In total, 41% of 408 municipalities (n = 169) reported at least one case. Within municipalities, there was a considerable heterogeneity in vaccination coverage and incidence by postal code area (Figure 2A and 2B).

Data on vaccination coverage by postal code area were obtained from the national vaccination register. MMR municipalities are those 29 municipalities where the early MMR vaccination campaign was conducted. In Vaals municipality in the far south-east of the Netherlands, a considerable number of infants receive their vaccinations in Germany and are therefore not included in the Dutch vaccination register. Eleven cases lacked location data and could not be included for measles incidence.

The incidence of reported cases by postal code area increased with a lower MMR-1 vaccination coverage (Figure 2C; Spearman’s correlation coefficient: -0.42).

**Figure 3:**
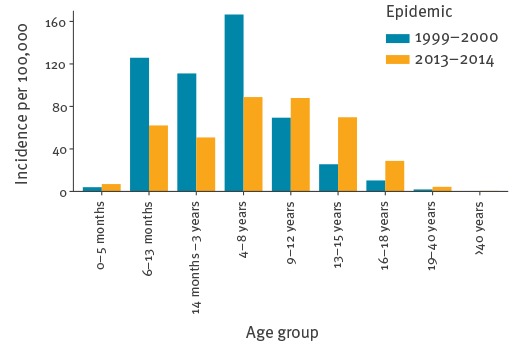
Incidence of reported cases by age group for the 1999–2000 epidemic (n = 3,170) and the 2013–2014 epidemic (n = 2,700), the Netherlands

The median age of reported cases was 10 years (range 0–68 years). Most reported cases were between 4 and 17 years of age (n = 2,092, 77%) (Table). Three per cent of the cases (n = 78) were under 14 months of age. Of these 78, three had been vaccinated once before onset of disease. Six cases were below 6 months of age (0.2%). Highest incidence rates were found in 4–8 year-olds and 9–12 year-olds (89 and 88 cases per 100,000, respectively) (Figure 3). Males and females were equally affected (1,355 of 2,684 cases where sex was known were female (50%)).

Age was calculated by subtracting date of birth from date of rash onset. For individuals older than 2 years, only the year of birth was known. For the purposes of the study, these cases were assumed to have been born on 30 June in the reported year of birth. For cases younger than 2 years, the month but not the day of birth was known, and so these cases were assumed to have been born on the 15^th^ day of the reported month of birth.

### Laboratory results

About a third of reported cases (n = 888, 33%) were laboratory-confirmed; all other cases were reported based on an epidemiological link. Most laboratory-confirmed measles cases (84%, n = 749) were confirmed using PCR testing of oral fluid or urine specimens. Another 13% (n = 116 cases) were confirmed by detection of measles-specific IgM antibodies in serum. In 2% of the cases (n = 16), both IgM and PCR test results were reported. For 1% of the cases (n = 7), the diagnostic test was not reported. Of the 749 PCR confirmed cases, 73% (n = 548) were sent to the national laboratory for sequencing. In 7% (n = 39) the sequence could not be identified, in 93% (n = 509), the sequence was identified as the D8 measles virus (MVs/Alblasserdam.NLD/22.13).

### Complications and hospitalisation

For 11% of the cases (n = 296) one or more complications were notified (Table). The occurrence of complications was unknown for 4% of the cases (n = 119). More than half of the cases with complications had pneumonia (54%) and about one third had otitis media (38%). The risk of complications was highest in cases below 4 years or above 40 years of age (both 16%). Otitis media was especially prevalent in children aged between 14 months and 3 years (6%). Pneumonia occurred most frequently in cases younger than 4 years of age (10%). Two reported cases were hospitalised with encephalitis: a 17-year-old girl and an 8-year-old boy. The girl had severe underlying medical conditions and died due to encephalitis and pneumonia.

Overall, seven per cent of the cases (n = 181) were hospitalised, most commonly for pneumonia (48%, n = 86) or dehydration/diarrhoea (15%, n = 27). For one per cent (n = 23), we do not know whether or not cases were hospitalised. Seven cases required intensive care admission for pneumonia (n = 5), encephalitis (n = 1) or both (n = 1). The median duration of stay in the hospital due to measles was 4 days (interquartile range 3–5 days). Adults with measles were at higher risk of hospitalisation than children (Table).

### Healthcare workers

In total, 19 HCW were reported to have acquired measles at work. Two of these were born before 1965 and were unvaccinated. Eight of the HCW with measles were born between 1965 and 1975, of whom only one was vaccinated (one dose). Of the four HCW born in 1975, 1976 and 1977 (these cohorts were offered only one vaccination during their childhood), three had been vaccinated once and one was unvaccinated. Five HCW were born after 1978, of whom two were unvaccinated and three had been vaccinated at least twice. Most infected HCW were working in a general practice (n=8) and three HCW acquired measles while working in a hospital. There were no reports of infected HCW transmitting measles to patients or other HCW, nor reports from patients infected while hospitalised.

### Comparison with the 1999–2000 epidemic

The 2013–2014 epidemic was comparable with the 1999–2000 epidemic in that it took place in the same low-vaccination-coverage areas and affected mostly the unvaccinated orthodox Protestant population. The age distribution of the epidemics, however, differed markedly (Figure 3). First, the median age in the 1999–2000 epidemic was 6 years [6], compared with 10 years in the recent epidemic (p value < 0.01).

Second, the incidence by age group of the two epidemics differed (p < 0.01). Older age groups (9 years and older) had a higher incidence in 2013–2014 than in 1999–2000, while the incidence in age groups below 9 years of age were halved in 2013–2014 compared with 1999–2000. Among infants aged 6–13 months, who were offered an early MMR vaccination in 2013–2014 but not in 1999–2000, the incidence in 2013–2014 was 62 per 100,000. This is significantly lower than the incidence of 126 per 100,000 reported in this age group in 1999–2000 (p < 0.05). In contrast, the incidence in infants below 6 months of age was higher in 2013–2014 than in the 1999–2000 epidemic (7 and 4 per 100,000, respectively) (p = 0.529).

## Discussion

Despite an MMR-1 vaccination coverage above 95% for the past 20 years in the Netherlands, a large measles epidemic of 2,700 reported cases, including cases with severe illness and one death, occurred in 2013–2014 among sociogeographically clustered orthodox Protestant communities with low vaccination coverage. The total costs of this epidemic were recently estimated at EUR 3.9 million [21].

In comparison with the previous epidemic in this group in 1999–2000, older age groups were more affected. There was a striking decline in reported cases during the summer holidays, which could be due to reduced transmission of measles and/or reduced reporting. The change of guidelines communicated by the RIVM to the MHS in mid-July 2013 to reduce the workload may also have influenced reporting.

The vast majority of reported cases were among unvaccinated orthodox Protestant individuals. The number of cases in other risk groups remained relatively low, which suggests limited contact with orthodox Protestants and more protection from herd immunity. Of the 141 vaccinated cases, most were in children between 4 and 8 years of age who had been vaccinated only once. Bringing the second MMR dose forward from 9-year-olds to 4-year-olds can reduce the susceptibility in this age group [22].

A limitation of our study is that it was based on reported cases only. After the 1999–2000 epidemic it was estimated that only 7% of all individuals with measles were reported [23]. Another study carried out a survey after the epidemic and identified 164 measles cases, of which only 9% (n = 15) had been reported during the 1999–2000 epidemic [24]. We found similar completeness of reporting of measles infections in this measles epidemic (data not shown). Based on this, the estimated number of individuals with measles infection in the 2013–2014 epidemic is ca 30,000. The use of non-invasive samples such as saliva and urine for measles diagnosis contributed to a higher proportion of infections being laboratory-confirmed or epidemiologically linked to a confirmed infection, and hence to a more complete reporting.

Eleven per cent of all reported cases had one or more complications. Similar to other epidemics [6,25-27], complications and hospitalisations were more likely to occur in young children and adults [17]. Cases with complications and/or hospitalisations were probably more likely to be reported than cases without complications, thus the true rate of complications and hospitalisations among all measles infections during this epidemic is likely to be lower than the 11% and 7% we found in reported cases, respectively.

A rare complication of measles, subacute sclerosing panencephalitis (SSPE), occurs months to years after measles infection. Recently, a case of SSPE was reported in a Dutch 17-year-old who died 4 months after diagnosis [28]. He had acquired measles in the Netherlands during the epidemic of 1999–2000 at the age of four years. SSPE is a very rare fatal complication of measles: estimates of SSPE incidence are ca 0.4–1.1 cases of SSPE per 10,000 cases of measles [29]. Assuming that 30,000 individuals acquired measles virus infection in the 2013–2014 epidemic, up to three cases of SSPE can be expected in the next two decades.

High measles vaccination coverage among HCW has been associated with decreased healthcare-associated measles virus infections among patients and personnel [30]. During this measles epidemic, 16 of 19 HCW with measles were incompletely vaccinated although they were eligible to complete their MMR vaccination schedule according to the advice of the OMT. An assessment of barriers to implementation of the recommendations is ongoing.

Compared with the previous epidemic in orthodox Protestants, we found a higher median age in the 2013–2014 epidemic and higher incidence rates in age groups above 8 years of age. This is likely due to the longer inter-epidemic interval before the 2013–2014 epidemic compared with the interval before the 1999–2000 epidemic [31]. The epidemic preceding the 1999–2000 epidemic was in 1992–1994, whereas the epidemic preceding the 2013–2014 epidemic was in 1999–2000. As a result, the susceptible population, consisting of individuals born since the previous epidemic, had a wider age range in 2013 than in 1999.

The cause of the lower incidence in children below nine years in the 2013–2014 epidemic compared with the 1999–2000 epidemic may be due to an increase in vaccination coverage among children under 9 years old in orthodox Protestant communities. Evidence for this was found in the serological surveys performed in 2006–2007 and 1995–1996, in which a higher proportion of diphtheria protection was found in the most recent survey [32]. Second, vaccination uptake in orthodox Protestants seems to be increasing generation on generation, as found in 2013 by assessing vaccination status of orthodox Protestants from the age of 18 to 40 years, their parents and their children (data not shown). Increasing vaccination coverage within these communities may also explain the longer inter-epidemic period [31] and, at least partly, the higher median age. The distribution of cases comprises a smaller proportion of young cases compared with the previous epidemic.

The lower incidence among infants 6–13 months of age could reflect the administration of early MMR vaccination. However, results are difficult to interpret given that the incidence was also relatively low in the adjacent older age groups. The incidence in infants aged less than 6 months was higher in 2013–2014 than in the 1999–2000 epidemic. This is likely to be related to the lower level of maternal antibodies in children born to vaccinated mothers compared with children born to unvaccinated mothers [16]. Measles vaccination began in the Netherlands in 1976. Therefore, in 2000, the proportion of infants born to vaccinated mothers was probably lower than in 2013.

The source of the first measles cases from this outbreak is unknown. According to the MEANS database, the Taunton sequence was first identified in Wales, UK, in the second half of 2012, and subsequently in many other cities in the UK throughout 2012 and the first half of 2013. At the time when the first Dutch case was identified with the Taunton sequence in May 2013, ca 900 identical sequences had been reported to MEANS, not only from the UK but several other countries within the WHO European Region (e.g. France, Ireland, the Russian Federation). Therefore, a particular source country is hard to identify [33,34]. The epidemic in the Netherlands, however, was indicated as the origin of outbreaks in Belgium [35] and Canada [36,37]. From Canada, onward transmission continued into the United States [38]. The likely spread to Belgium led to an outbreak in a day care centre with 33 reported cases. In Canada an outbreak took place in Alberta with 43 reported cases and another in British Columbia with 444 reported cases. Social ties exist between orthodox Protestants in the Netherlands and Canada and the spread of infections such as poliomyelitis, measles, mumps, and rubella to Canada has been reported before [39].

Improved vaccination coverage among orthodox Protestants is essential to prevent future outbreaks. It is therefore one of the prioritised interventions in the Netherlands’ national measles elimination plan [40]. Since orthodox Protestants base their vaccination decisions largely on religious arguments [41], specific information materials were developed focusing on religious arguments for and against vaccination. These brochures aim to facilitate decision making about vaccination among orthodox Protestants and were distributed during the epidemic [42]. An evaluation of their acceptability and impact is currently ongoing.

Vaccination coverage seems to be increasing within the orthodox Protestant community. An improvement in vaccination coverage will be reflected in a different epidemiology of future epidemics. In the current epidemic, a longer inter-epidemic period resulted in older age groups affected in comparison to the previous epidemic.

The number of individuals refraining from vaccination is insufficient to sustain endemic measles transmission in the Netherlands. Nevertheless, this situation does pose a risk to public health in the Netherlands and contributes to the worldwide spread of measles, thus forming an impediment to the elimination of measles in Europe and elsewhere.
